# Rapid, Time-Division Multiplexed, Direct Absorption- and Wavelength Modulation-Spectroscopy

**DOI:** 10.3390/s141121497

**Published:** 2014-11-14

**Authors:** Alexander Klein, Oliver Witzel, Volker Ebert

**Affiliations:** 1 Physikalisch-Technische Bundesanstalt, Bundesallee 100, Braunschweig 38116, Germany; E-Mails: alexander.klein@ptb.de (A.K.); oliver.witzel@ptb.de (O.W.); 2 Center of Smart Interfaces, TU Darmstadt, Petersenstraße 32, Darmstadt 64287, Germany

**Keywords:** trace gas measurement, absorption spectroscopy, TDLAS, WMS, hygrometer, calibration-free

## Abstract

We present a tunable diode laser spectrometer with a novel, rapid time multiplexed direct absorption- and wavelength modulation-spectroscopy operation mode. The new technique allows enhancing the precision and dynamic range of a tunable diode laser absorption spectrometer without sacrificing accuracy. The spectroscopic technique combines the benefits of absolute concentration measurements using calibration-free direct tunable diode laser absorption spectroscopy (dTDLAS) with the enhanced noise rejection of wavelength modulation spectroscopy (WMS). In this work we demonstrate for the first time a 125 Hz time division multiplexed (TDM-dTDLAS-WMS) spectroscopic scheme by alternating the modulation of a DFB-laser between a triangle-ramp (dTDLAS) and an additional 20 kHz sinusoidal modulation (WMS). The absolute concentration measurement via the dTDLAS-technique allows one to simultaneously calibrate the normalized 2f/1f-signal of the WMS-technique. A dTDLAS/WMS-spectrometer at 1.37 μm for H_2_O detection was built for experimental validation of the multiplexing scheme over a concentration range from 50 to 3000 ppmV (0.1 MPa, 293 K). A precision of 190 ppbV was achieved with an absorption length of 12.7 cm and an averaging time of two seconds. Our results show a five-fold improvement in precision over the entire concentration range and a significantly decreased averaging time of the spectrometer.

## 1. Introduction

Tunable diode laser absorption spectroscopy (TDLAS) is an outstanding technique for concentration and temperature measurements of gaseous species in scientific as well as industrial applications. It is used in combustion diagnostics where its minimally invasive, sampling-free approach and the high temporal resolution are especially appreciated [1–5]. Further, TDLAS is used in environmental and process monitoring for absolute and calibration-free detection of multiple gas species, e.g., H_2_O, CO_2_ or CH_4_ [6–9]. Direct TDLAS (dTDLAS) and WMS are the preferred techniques used in laser absorption spectroscopy. Both techniques use a monochromatic beam of a tunable diode laser to spectrally scan over a molecular absorption line. dTDLAS uses a single wavelength scan to retrieve direct transmission spectra and derive absolute gas concentrations. In contrast, WMS applies an additional sinusoidal modulation in the kHz-range on top of the scan to allow phase sensitive detection of the higher harmonics via lock-in techniques. The dTDLAS approach has the advantage to be calibration-free [10]. Harmonic WMS detection usually allows higher sensitivity due to reduced 1/f noise, but requires a sensor calibration.

Some approaches have tried to combine the benefits of dTDLAS and WMS. Duffin *et al.* recovered direct absorption line shapes from the first harmonic WMS signal [11]. However, this technique is designed for measuring high concentration levels for process control, where the modulation depth for an optimum signal to noise ratios (SNR) doesn't have to be maximized. Lins *et al.* investigated the impact of analog-digital converter resolution on both techniques by slow successive changing between WMS and dTDLAS [12]. A calibration-free WMS model was developed by Rieker *et al.* [13], but this technique requires extensive laser characterization to obtain full WMS line shapes. Therefore one major use is a fixed wavelength WMS mode to receive gas species information by simulating 2f spectra against 2f peak measurements. This technique can be beneficial in harsh environments, whereas fitting full line spectra would lead to more absolute information about the measurement result. Recently, Sun *et al.* and Goldenstein *et al.* developed a method to simulate WMS spectra and subsequently compare the simulation with the measurement to infer gas properties without calibration [14,15]. This method shows good results for the studied high pressure and temperature environments. However, this method relies on an extensive laser characterization. This characterization should display the exact wavelength modulation shape and amplitude. The nonlinear amplitude of the sinusoidal modulation over the entire wavelength scan should lead to strong effects in the 2f line shape, especially for large wavelength scans. These nonlinear effects have up to now not been discussed or quantified.

In this paper we want to combine the accuracy of the calibration-free dTDLAS with the enhanced precision of the WMS-2f by quasi-simultaneously using both spectroscopic techniques with a new rapidly time-division multiplexed modulation scheme. It is important to note that our WMS- and dTDLAS variants are based on an identical hardware setup. A wavelength modulated laser beam is scanned over an absorption feature of the target molecule. The resulting transmission signal is collected by a photodiode and subsequently digitized for further analysis. Hence, the two techniques can be combined to achieve absolute calibration-free gas species concentrations with high sensitivity. The fundamental approach in this work is to simultaneously use the absolute, calibration-free concentration obtained by dTDLAS for a direct, on-the-fly calibration of the WMS signal with higher sensitivity in a time division multiplexed procedure. This approach was realized by building a spectrometer to prove the advantage of simultaneously coupling and using both spectroscopic techniques for concentration measurements.

## 2. Spectroscopic Techniques

The basic principles of dTDLAS and WMS are well described in literature [16–20]. For reasons of continuity they will be briefly discussed and important points for this paper will be emphasized.

### 2.1. Direct Tunable Diode Laser Absorption Spectroscopy (dTDLAS)

dTDLAS uses a high spectral resolution, continuously wavelength tunable diode laser as light source. The transmitted light is attenuated by wavelength-dependent molecule specific absorption, while the laser wavelength is periodically scanned by a triangular laser current modulation. The repetition rate of this modulation is chosen depending on the application requirements and is typically in the range between 100 Hz and 10 kHz [21]. The transmission *T(v)* can be described by the extended Lambert–Beer Equation (1) which includes background radiation (*E(t)*) and spectrally broadband transmission losses (*Tr(t)*):

(1) $$\text{T}\left(\text{v}\right)=\frac{\text{I}\left(v\right)}{{\text{I}}_{0}\left(v\right)}=\text{Tr}\left(\text{t}\right)\cdot {\text{e}}^{-\text{S}\left(\text{T}\right)\cdot \phi \left(v-v_{0}\right)\cdot \text{N}\cdot \text{L}}+\text{E}\left(\text{t}\right)$$

This can be converted to extract the H_2_O volume mixing ratio, *c*, Equation (2) by applying the ideal gas law:

(2) $$c=-\frac{k_{B}\cdot T}{S\cdot L\cdot p}\int^{\;}\ln\left(\frac{I\left(v\right)-E\left(t\right)}{I_{0}\left(v\right)\cdot Tr\left(t\right)}\right)\frac{dv}{dt}dt.$$

here *S* denotes the temperature dependent molecular linestrength of the used H_2_O transition, *Φ*(*ν*−*ν_0_*) the line shape function, *N* the absorber number density, *k_B_* the Boltzmann constant, *T* the measured gas temperature, *L* the absorption path length, *I*(*ν*) the measured intensity at the detector, *I_0_*(*ν*) the initial light intensity, *E*(*t*) the background emission and *Tr*(*t*) the broadband transmission losses. Finally, *dν*/*dt* describes the dynamic tuning behavior of the laser, which has to be determined experimentally detecting the Airy-signal when the laser light is transmitted through a planar air-spaced etalon. A closer look at Equation (2) shows that no additional calibration parameter is needed to derive the absorber density. Hence by simultaneously measuring pressure and temperature, absolute species concentrations can be obtained without calibration against a concentration-reference, which is why we call this technique calibration-free. The absolute accuracy of this calibration-free technique has lately been demonstrated by a metrological comparison against a primary H_2_O reference standard to be on the order of 0.3% [10].

### 2.2. Wavelength Modulation Spectroscopy

WMS utilizes a more complex laser modulation to generate higher harmonics of the absorption signal, thereby shifting the measured signals to higher frequencies to reduce 1/f intensity noise *via* phase sensitive detection e.g., lock-in amplifier. Here, fast sinusoidal modulation in the kHz-range is superimposed on the slower modulation with a linear slope as used in dTDLAS. Due to the nonlinear nature of the molecular absorption lineshape, higher harmonics are generated at multiple frequencies of the fundamental sinusoidal modulation frequency. The time dependent wavelength of the laser can be described as:

(3) $$v\left(t\right)=v_{0}(t)+a\;\cos\left(\omega t\right)$$

here *v*_0_ is the center frequency of the laser, *a* the modulation amplitude and *ω* = 2*πf* is the angular frequency. The transmission signal *I(t)*/*I_0_(t)* can be expanded in a Fourier series:

(4) $$I(t,v)=I_{0}(t,v)\cdot \left(v_{0}\left(t\right)+a\;\cos\left(\omega t\right)\right)=I_{0}(t,v)\cdot \sum_{n=0}^{n=\infty }H_{n}\left(v,a\right)\;\cos\left(n\omega t\right)$$

For small absorbances (smaller 0.1) Equation (1) can be simplified to:

(5) $$T\left(v\right)=\frac{I\left(v\right)}{I_{0}\left(v\right)}=1-S\left(T\right)\cdot \phi \left(v-v_{0}\right)\cdot N_{V}\cdot L=1-k\left(v\right)\cdot L.$$

with *k(υ)* denoting the absorption coefficient. Using the expression derived by Arndt [22] the amplitudes of the higher harmonics can be described by:

(6) $$\begin{array}{c} \begin{array}{cc} H_{n}\left(m,x\right)=\frac{{ɛ}_{n}}{2}\frac{i^{n}}{m^{n}}\cdot \frac{{\left[\sqrt{{\left(1-ix\right)}^{2}+m^{2}}-\left(1-ix\right)\right]}^{n}}{\sqrt{{\left(1-ix\right)}^{2}+m^{2}}}+c.c. & {ɛ}_{n}=\{\begin{array}{cc} 1; & n=0\\ 2; & n=1,2,3\ldots \end{array} \end{array}\\ \begin{array}{cc} m=\frac{a}{\gamma }\;; & x=\frac{v}{\gamma } \end{array} \end{array}$$

here *m* denotes the modulation depth relative to the absorption linewidth γ. In this description, the effects introduced by the non-linear characteristics of the diode laser modulation or optical components are neglected. These background signals have been analyzed in detail by Kluczynski *et al.* [23]. Equations (5) and (6) showing that the harmonic signals are linearly proportional to the absorption caused by the gas species, the absorption path length, the initial light intensity and the modulation index *m*. Usually the first and second harmonic signal is demodulated using a lock-in amplifier to achieve almost background free measurements. Often the 2f signal is normalized by the 1f signal to correct transmission fluctuations [24–26]. Residual amplitude modulation (RAM) never the less induces background signal, due to the nonlinear intensity properties of real world laser diodes. This RAM can be corrected by prior system measurement in the absence of absorption. For measurements at constant pressure the amplitude of the first and second harmonic are maximized for *m* = 2.2 and hence the detector signal is optimized. Using lock-in detection higher harmonic signals can be extracted. The extracted WMS-2f signal is proportional to the gas concentration and needs to be calibrated to achieve absolute concentration measurements.

## 3. Experimental

### 3.1. Set Up

**Figure 1.**
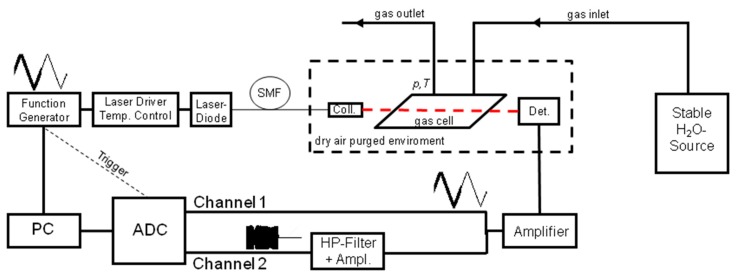
Experimental setup for the rapid time-division multiplexed direct absorption and wavelength modulation spectrometer.

To take advantage of the calibration-free approach of dTDLAS and the effective noise suppression of WMS without sacrificing the calibration free capabilities a spectrometer was built to quasi simultaneously combine both techniques via rapid time multiplexing. The spectrometer setup is shown in Figure 1. A fiber coupled distributed feedback diode (DFB) laser (NEL America Inc., Saddle Brook, NJ, USA) with a laser driver and temperature controller (Thorlabs Inc., Newton, NJ, USA) was used to scan over a single absorption line (211←110) of H_2_O at 1369.97 nm. It should be mentioned that the wavelength scanning range of DFB diode laser can decrease strongly for higher modulation frequencies [21]. Therefore it is not straight forward and advantageous to implement calibration-free dTDLAS at the same measurement frequency as WMS-2f to have the same level of 1/f noise reduction, as the entire absorption line has to be covered. An arbitrary function generator (Teledyne LeCroy GmbH, Heidelberg, Germany) applies a time division multiplexed (125 Hz) triangle- and a superimposed 20 kHz sinusoidal modulation for dTDLAS and WMS, respectively. The amplitude of the sinusoidal modulation is chosen to maximize the 2f signal corresponding to a modulation index of *m* = 2.2. The collimated laser beam is guided through a single path gas cell (*L* = 12.7 cm) and detected by an InGaAs photodiode (Hamamatsu Photonics, Hamamatsu, Japan). Neither for dTDLAS nor for WMS is a line-locking technique used to minimize the experimental complexity. The windows of the gas cell are tilted by 45° to avoid back reflections, which can cause interferences, so called fringes, on the signal. Using a humidity generator (Thunder Scientific Cooperation, Albuquerque, NM, USA) a humidified air flow of 3 slpm with highly stable H_2_O concentration is generated and passed through the absorption cell. Gas pressure and temperature are logged every three seconds during the experiment (Omega Newport, PT-100). The beam path outside the cell is placed in an enclosed environment and purged with dry air with residual moisture of less than 3 ppmV to avoid systematic offsets in the H_2_O absorption signal [27].

**Figure 2.**
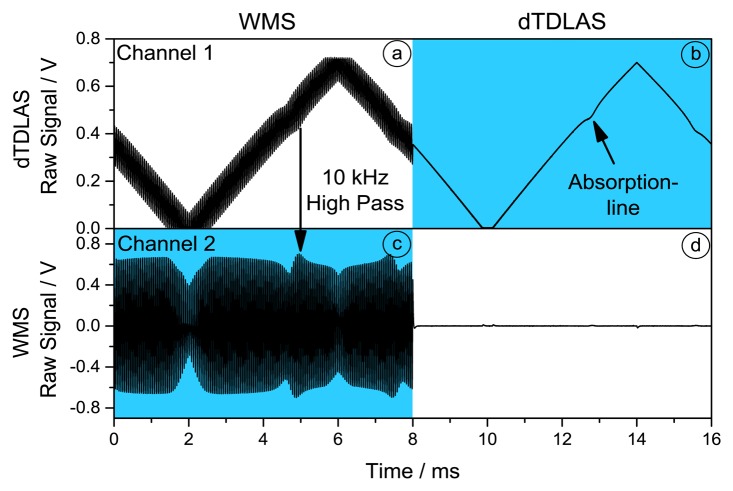
Digitized raw signals of dTDLAS and WMS channels with 10 MS/s and 14 bit resolution. Top row: Raw signal for WMS (**a**) and dTDLAS (**b**). Bottom row: High pass filtered WMS signal (**c**) and dTDLAS signal. Only the highlighted signals ((b) and (c)) are used for later analysis. The high passed signal in channel 2 is used to extract the 1f and 2f component for the WMS analysis (b).

The detector photocurrent is converted into a voltage signal by a low-noise transimpedance amplifier (TIA). The voltage signal is split and one part corresponding to the dTDLAS signal is directly digitized with 10 MS/s at 14 bit. The WMS signal on the second part is first high pass filtered and amplified at a cut off frequency of 10 kHz to fully utilize the dynamic range of the analog digital converter (ADC). The digitized raw signals are shown in Figure 2, where the highlighted parts are used for the later analyses. dTDLAS does not require the high sample resolution of 10 MS/s, hence the number of signal points are reduced by block averaging 40 consecutive samples along the absorption profile before the fit procedure.

**Figure 3.**
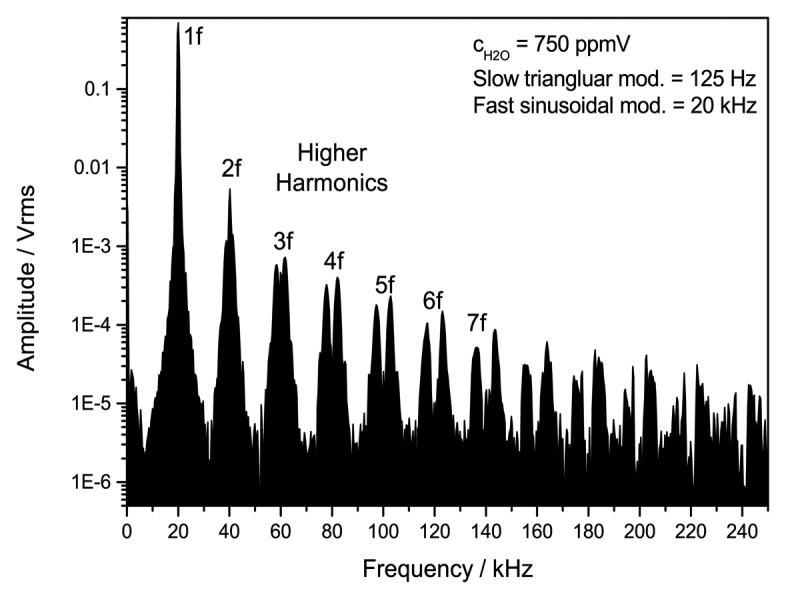
Experimental frequency spectrum of the digitized, high-pass filtered photodiode raw signal measured for 750 ppmV H_2_O. For WMS a 125 Hz linear triangular modulation is used to scan over the absorption line. This scan is superimposed with a 20 kHz sinusoidal modulation.

WMS is analyzed using the digitized, high-passed signal of channel 2. A digital lock-in detection scheme is applied using LabVIEW which enables the demodulation of several harmonic signals at the same time. Figure 3 shows a frequency spectrum of the digitized WMS raw signal with several higher harmonics of the absorption signal.

The peak amplitude strongly decreases for higher order harmonics. Hence, care must been taken when demodulating higher order harmonics to avoid finite ADC resolution effects which decrease the retrieval quality. The 1f and 2f signal is demodulated by multiplying the digitized signal by cosines and sines functions with a frequency of 20 kHz and 40 kHz, respectively and subsequent low pass filtering. The phase is adjusted to zero the X-component of this digital lock-in. Hence the entire 2f signal is demodulated in the Y-component. A digital Butterworth filter with a cut off frequency of 2.3 kHz is applied corresponding to a time constant of 69.2 μs of the digital lock-in amplifier. As the aim of this work is using both spectroscopic techniques to increase the precision and the dynamic range of absorption measurements no scan averaging is applied at any time in the results shown below.

### 3.2. WMS – Calibration Procedure

**Figure 4.**
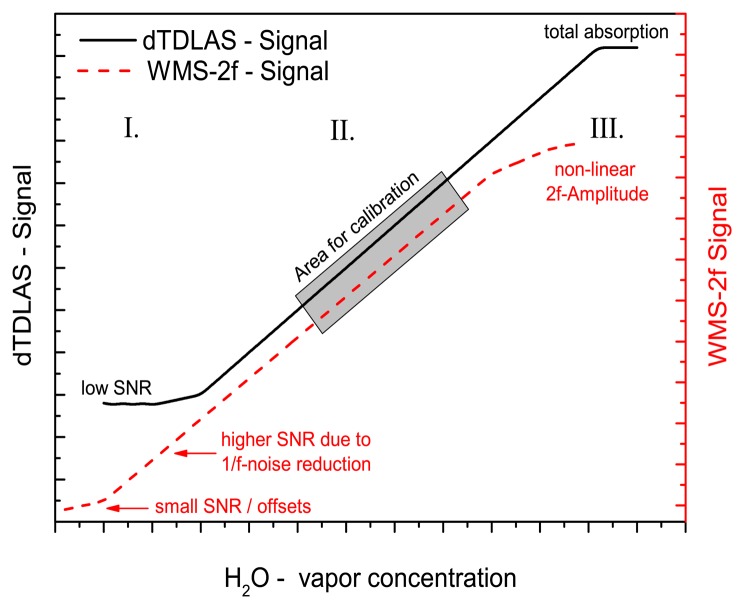
Schematic of the *on-the-fly* WMS calibration using quasi simultaneous rapid time multiplexed dTDLAS/WMS measurements.

Figure 4 schematically illustrates how the two spectroscopic techniques are combined for WMS calibration. When the molecular absorbance (*ln(I*/*I_0_)*) is above 0.1 solely the dTDLAS signal is used as the WMS signal at high absorptions scales nonlinear with H_2_O concentration. In the absorbance range below 0.1 the normalized 2f/1f WMS signal is calibrated against the absolute concentration gained from dTDLAS with a sufficient SNR.

In this region the 2f/1f signal can be used to increase the precision of the concentration measurement, due to the higher SNR of the WMS signal. With further decreasing absorbance the SNR of the dTDLAS gets to small and the calibrated WMS signal with a higher SNR is used to derive the concentration. For this we first fit the normalized WMS-2f/1f signal against a reference WMS trace using linear regression. The derived slope of the fit is directly calibrated against the calibration-free derived H_2_O-concentration using dTDLAS. For instance, at the beginning of a concentration measurement only a single calibration point is available. By decreasing or increasing the species concentration more and more points are added to the calibration curve. Hence the calibration gets more accurate. This continues until the SNR of the dTDLAS is too small or the absorbance is above 0.1. This could be especially an advantage for in-field trace gas measurements as our new approach calibration can be conducted continuously on the fly, without any need for reference gases or measurement interruption. The individual steps are explained in more detail below.

## 4. Analysis of dTDLAS and WMS-2f/1f

In this section the analysis of the individual, interleaved, rapidly, time-division multiplexed dTDLAS and WMS will be discussed further in detail. First, the dTDLAS measurements are shortly explained as they serve as “calibration reference” for the WMS measurement. Secondly, the analysis of the higher SNR WMS signal to obtain a relative concentration is reported. Finally, the simultaneously acquired dTDLAS and WMS measurements are compared and combined to transfer the WMS signal on an absolute H_2_O scale.

### 4.1. dTDLAS Measurements

**Figure 5.**
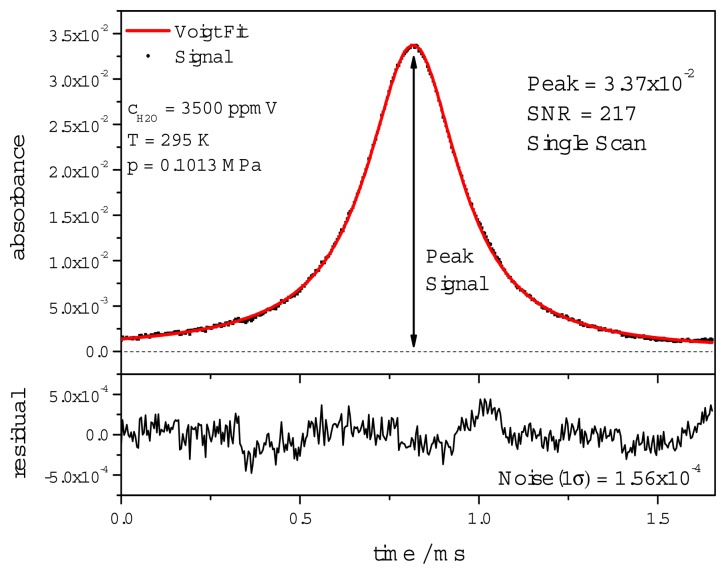
Single scan (simultaneously to WMS acquired) dTDLAS line profile at 3500 ppmV. The H_2_O concentration is obtained using a nonlinear Levenberg-Marquardt multi-line Voigt fit algorithm. The residuum is shown below with an absorbance noise of 1.56 × 10^−4^ resulting in a SNR of 217.

The model function used for dTDLAS scan fitting is composed of a third order polynomial function for the baseline and a Voigt line shape function for the absorption line. The line width is calculated from broadening coefficients based on HITRAN [28] and measured gas pressure and temperature in order to minimize the degrees of freedom of the fit. Figure 5 shows a typical single dTDLAS scan at 3500 ppmV with an absorbance of 3.4% and a SNR of 217.

here the SNR is defined as the peak absorbance of the absorption line divided by the standard deviation of the fit residuum. The obtained H_2_O concentration is derived without prior calibration of the system and is subsequently used to calibrate the WMS-2f/1f signal.

### 4.2. WMS Measurements

**Figure 6.**
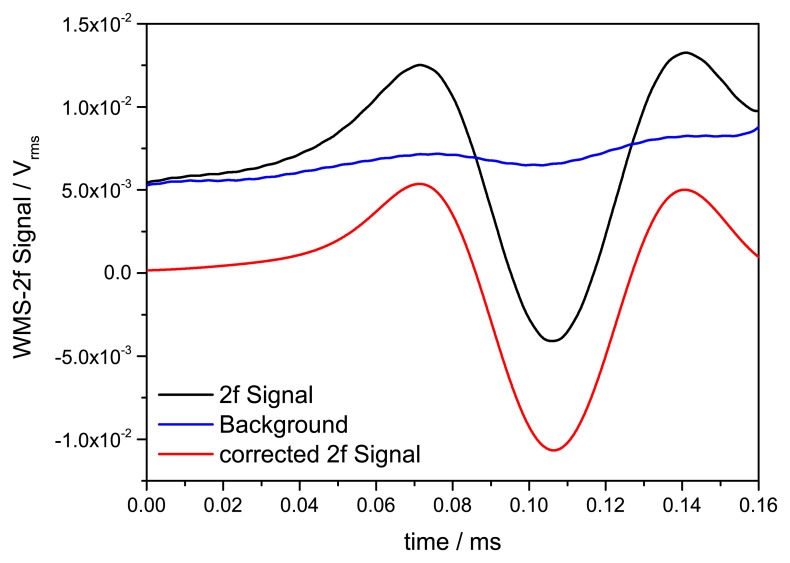
The demodulated 2f signal and corrected 2f signal after and before background subtraction.

**Figure 7.**
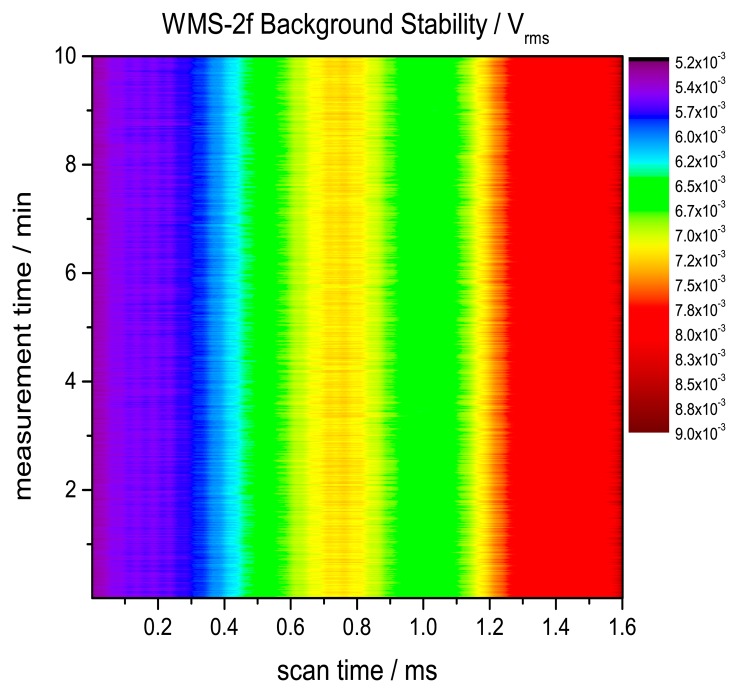
Measured time series of the background caused by RAM. The time series shows a very stable background with minor fluctuation basically caused by electronic noise with a standard deviation of 5.3 × 10^−5^ V_rms_.

To derive 2f signal the WMS raw signal is demodulated at 40 kHz (Figure 6). Our measured 2f signal shows an additive background caused by residual amplitude modulation (RAM) due to the nonlinear intensity modulation of the laser. To correct this background, measurements in the absence of H_2_O absorption were conducted. To reduce minor fluctuations of this background correction a 100 times scan averaged signal was used for subsequent background subtraction. A time series of the background measurement was taken to verify a stable RAM background without drifts due to the laser or electronic instability. Figure 7 shows the stability of the background measurement for a time span of 10 min. No drifts can be observed. Fast minor background fluctuations in the order of 5.3 × 10^−5^ V_rms_ are caused by electronic noise.

**Figure 8.**
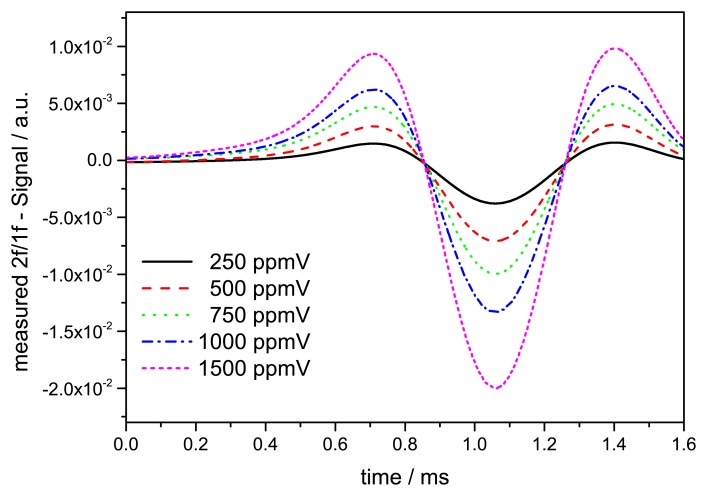
WMS-2f signal for concentration steps from 250 ppmV up to 1500 ppmV.

After background correction the WMS-2f signal is normalized to the simultaneously demodulated 1f signal to account for transmission fluctuations. Figure 8 displays the measured WMS-2f/1f signal for concentration steps from 250 ppmV to 1500 ppmV indicating the linear response of the normalized WMS-2f/1f peak to the concentration level.

The normalized 2f/1f profile is fitted against an experimental reference H_2_O profile using a linear regression model [29]. This step is done to use the entire information of the absorption profile instead of using spectral one point measurements like in fixed wavelength WMS. The reference profile is a 100 times average to reduce fluctuation.

**Figure 9.**
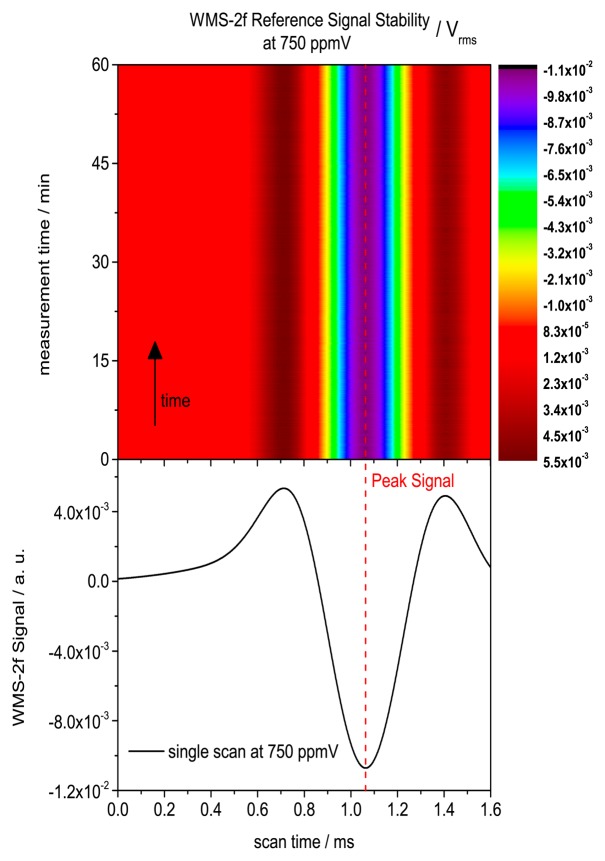
Time series measurement of reference spectra at 750 ppmV over 60 min to assure stability, where the 2f-amplitude is color coded. Below a single corresponding WMS-2f scan is shown. The measurement was performed at 125 Hz. But only three scans per second were saved to save computer memory. The achieved standard deviation of the peak WMS-2f signal was 5.2 × 10^−5^ V_rms_.

As for the background it is important to assure stability of the reference spectra. Here, in contrast to the background, the stability of the humidity reference source is important. For this reason a time series at 750 ppmV over 1h was measured to analyze and exclude any laser or concentration drifts. Figure 9 clearly displays the stability of the reference source as well as the spectrometer stability.

The achieved standard deviation of the WMS-2f signal was 5.2 × 10^−5^ V_rms_. This small fluctuation is mainly caused by electronic noise. However, this fluctuation would correspond to a concentration change of 3.6 ppmV or 0.5% change at the measured concentration level at 750 ppmV. The standard deviation of the 100 times averaged signal decreases to 1.2 × 10^−5^ V_rms_ corresponding to a concentration fluctuation of 0.81 ppmV or 0.1% at 750 ppmV.

**Figure 10.**
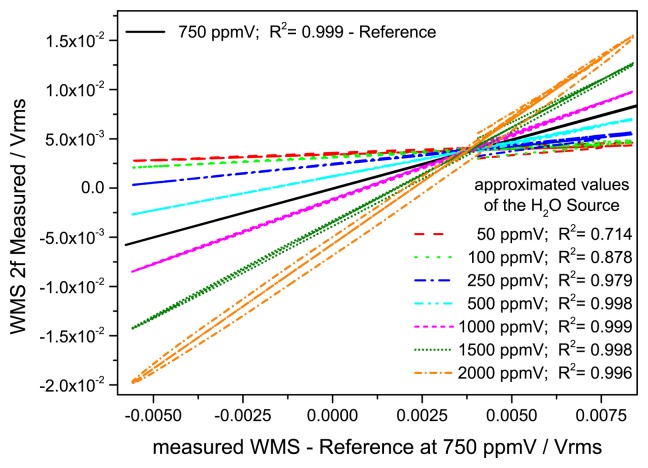
Correlative linear fits of the measured 2f/1f spectra for several H_2_O concentration levels against a reference spectrum at 750 ppmV.

Figure 10 shows the fits gained by the linear regression procedure against a reference spectrum at 750 ppmV and their residuals. For concentrations smaller than the reference the slope is smaller and for higher concentrations bigger than unity. The extracted slope of this linear regression is then calibrated against the absolute concentration derived from the quasi simultaneous dTDLAS measurement. With increasing H_2_O concentration the slope wings are shifting further apart due to the increased self-broadening of the absorption line. This results in a larger deviation for the linear fit, but this does not affect the derived slope with the H_2_O concentration. For further improvement of this regression procedure more reference spectra at different concentration levels could be used.

## 5. Results and Discussion of the Time Multiplexed Spectroscopic Scheme

**Figure 11.**
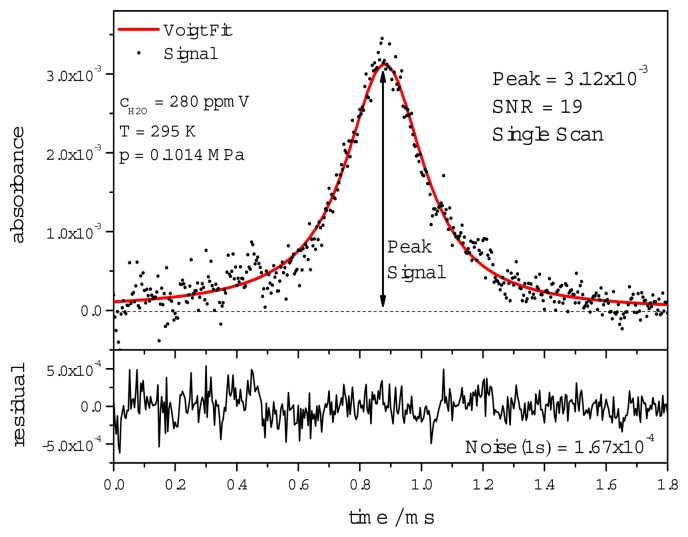
Typical single dTDLAS scan and fit (red) acquired at 280 ppmV H_2_O in air. The fit residuum is shown below with an absorbance noise of 1.67 × 10^−4^.

To validate the spectroscopic scheme, measurements over the range of 50–3500 ppmV H_2_O in air were carried out using the experimental setup described in Section 3.1. A typical dTDLAS scan at 280 ppmV (T = 295 K, p = 0.1014 MPa) with a peak absorbance of 0.31% is shown in Figure 11 where the electronic noise clearly influences the measurement quality resulting in a SNR of 19.

**Figure 12.**
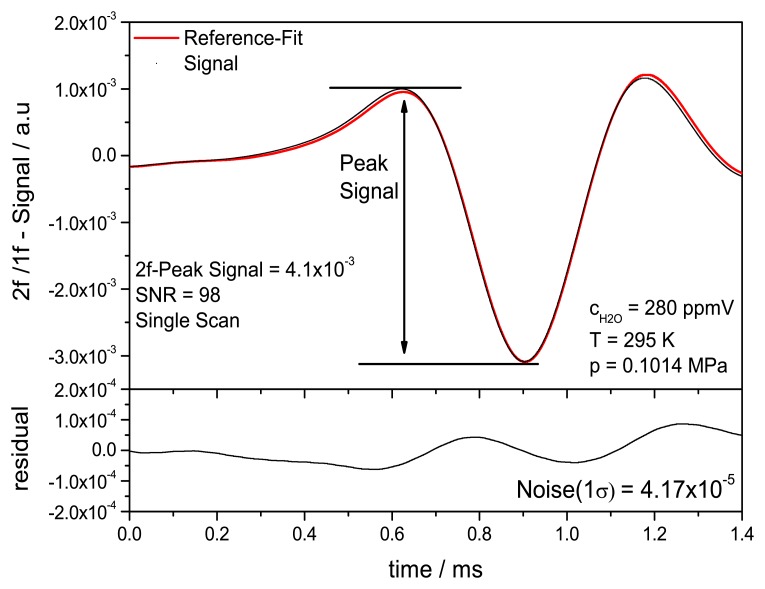
Single WMS 2f/1f scan measured at 280 ppmV H_2_O using a 125 Hz triangle modulation with a 20 kHz superimposed sinusoidal. A correlation fit to a WMS reference scan at 750 ppmV is applied.

Figure 12 shows the quasi-simultaneously acquired single scan normalized WMS-2f/1f scan at a H_2_O concentration of 280 ppmV in air (T = 295 K, p = 0.1014 MPa). The fit is realized using the linear regression against reference spectra at 750 ppmV as explained above. A SNR of 98 was obtained and a standard deviation of 4.17 × 10^−5^. Here the SNR for the WMS-2f/1f measurement is defined as the peak to peak difference divided by the standard deviation of the residual. Fitting routines for WMS and dTDLAS were implemented using LabVIEW. Comparing the single shot WMS to the simultaneous acquired single shot dTDLAS signals shows a fivefold increase of SNR. This illustrates the benefit using the time division multiplexed dTDLAS-WMS detection scheme. The SNR is currently mainly limited by the deviation of the correlative fit, hence the SNR could be further optimized by referencing to multiple scans at different concentrations or even further by an improved fitting procedure.

**Figure 13.**
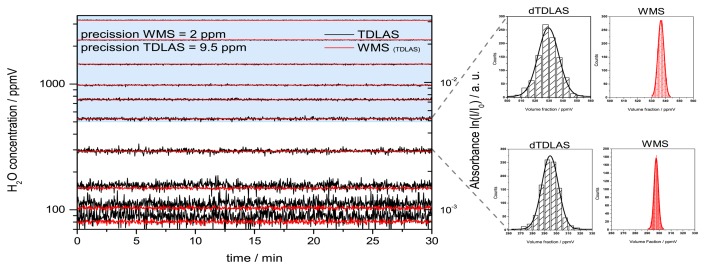
Left side: quasi simultaneous, multiplexed dTDLAS and WMS-2f measurement for several 30 min concentration steps. The blue highlighted area is used to calibrate the WMS-2f/1f signal using dTDLAS. Right side: The measurement noise are clearly Gaussian distributed (right panels), where the WMS results show a fivefold smaller half width of the distribution resulting in better precision in concentration of 2 ppmV.

To investigate the linearity and resolution of the multiplexed dTDLAS/WMS scheme, measurements at several H_2_O concentrations levels were conducted with a measurement time of 30 min per level. Each concentration level was measured after adjusting the humidity generator and awaiting stable source conditions (Figure 13).

**Figure 14.**
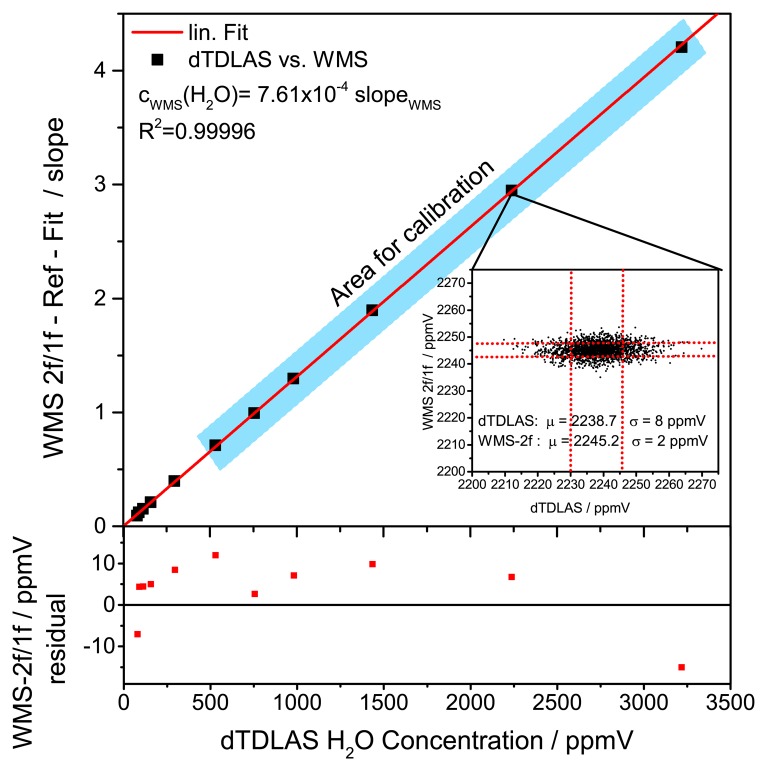
WMS calibration of the normalized WMS 2f/1f measurements against the absolute H_2_O concentration obtained using dTDLAS. The excellent linearity of WMS and dTDLAS measurements results in a *R*^2^ of the calibration of 0.99996. The insert shows single scan results over a 30 min long concentration step.

The WMS signal was first fitted against a 750 ppmV reference profile and the fitted slope was subsequently calibrated using the H_2_O volume fraction obtained by dTDLAS. The minimum SNR of the dTDLAS signal to be used for calibration was chosen to SNR 20 (Figure 14). Hence the range for direct calibration of the WMS signal against the dTDLAS is from 500 ppmV to 3500 ppmV (blue highlighted in Figure 14). Mean values and standard deviation of the individual concentration levels corresponding to the spectrometer precision were calculated and compared. The results are summarized in Table 1. For the dTDLAS measurements a constant precision around 9 ppmV was achieved. Results obtained using WMS show a precision of approximately 2 ppmV. Hence, the relative deviation corresponding to the species concentration divided by the standard deviation is 1.8% on average and does not exceed 3% over the measured concentration range.

The histograms in Figure 13 show nice Gaussian distributions. The half width half maximum (HWHM) of the Gaussian profile correspond to the measurement precision of 9.5 ppmV (dTDLAS) and 2 ppmV (WMS), which corresponds also to the standard deviation of this H_2_O level. It should be mentioned that the stability and precision of these concentration measurements are also affected by the humidity generator stability which is specified by the manufacturer to be about 0.1% relative to the ppmV level. The WMS-2f/1f measurements display clearly an enhanced precision compared to dTDLAS. This enables concentration measurements with an increased precision and makes measurements at lower concentration levels possible, where dTDLAS measurements have a smaller SNR due to a higher noise level.

**Figure 15.**
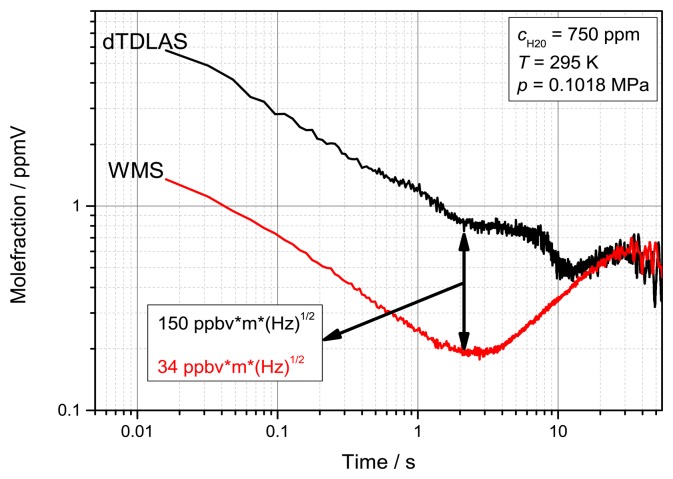
Allan deviation the time division multiplexed dTDLAS and WMS measurement at *c*_H2O_ = 750 ppmV. The normalized 2f/1f WMS need less averaging to receive a normalized precision of 34 ppbV·m·(Hz)^1/2^.

To further compare the two spectroscopic techniques a calculation of the Allan deviation of a simultaneously measured concentration of 750 ppmV was conducted (Figure 15). The optimal precision for the time multiplexed measurements was achieved with the WMS signal already after 2 s (125 scans) of averaging and resulted in a normalized sensitivity of 34 ppbV·m·(Hz)^1/2^. After 1 s the dTDLAS approach shows a four times worse normalized precision of 150 ppbV·m·(Hz)^1/2^. The Allan deviation also indicates that the lowest stable precision for the WMS measurements needs less averaging time. WMS could therefore be used to increase the time resolution of calibration-free absorption measurements. After 10 s averaging both measurements show a similar unstable deviation, which we interpret as fluctuations and drifts of the humidity generator, probably caused by the control loop.

## 6. Conclusions and Outlook

A new, rapidly time-multiplexed, direct absorption and wavelength modulation-spectroscopy scheme has been developed, evaluated and used to build an absolute calibration-free, wide range WMS-dTDLAS spectrometer. The rapidly alternating modulation between two spectroscopic techniques enables simultaneous detection of absolute H_2_O concentration via dTDLAS and higher SNR H_2_O concentration measurements via the WMS in a time-division multiplexed manner. The normalized second harmonic spectrum of the WMS signal is fitted using linear regression against a reference spectrum. The slope of the fit is directly calibrated to the absolute concentration measurement of the dTDLAS signal with sufficient SNR. This offers the possibility of calibration-free species concentration measurements with enhanced precision and dynamic range, due to 1/f-noise reduction. At least five times improvement of SNR and precision of H_2_O concentration measurements in air could be achieved in the range of 50–3000 ppmV by the dTDLAS calibrated WMS.

The newly developed detection scheme combining the two widely used techniques dTDLAS and WMS could significantly improve species concentrations measurements in several areas, such as atmospheric trace gas detection. Further with increased precision and SNR smaller concentration levels could be detected or absorption length could be decreased offering a new possibility for minimal invasive sensors.

## Figures and Tables

**Table 1.** Standard deviations during stable H_2_O concentration step measurements.

***C*_H2O_/ppmV**	**σ_dTDLAS_**	**σ_WMS_**
79.3	8.9	1.90
89.0	9.6	2.0
112.0	8.4	2.0
156.5	8.2	1.9
294.5	8.9	1.7
529.4	9.3	2.2
754.3	8.9	1.6
980.2	7.9	2.0
1440.0	8.9	1.9
2240.0	8.7	1.6
3220.0	8.2	1.4
